# Speciation by hybridization: the mind-boggling nature, educational, and research value of the largest group of unisexual vertebrates

**DOI:** 10.1093/biosci/biaf010

**Published:** 2025-02-14

**Authors:** Anthony J Barley, Charles J Cole

**Affiliations:** School of Mathematical and Natural Sciences, Arizona State University–West Valley, Glendale, Arizona 85306, United States; Department of Herpetology, American Museum of Natural History, New York, New York 10024, United States

**Keywords:** parthenogenesis, polyploidy, evolution, speciation, *Aspidoscelis*

## Abstract

Thirteen species of North American lizards are remarkable because only females exist, which reproduce by cloning unfertilized eggs. Their closest relatives reproduce sexually, with eggs fertilized by sperm from males, as in most vertebrates. The unisexual species originated through hybridization, dispensing with sex and males in a single generation. These lizards hold tremendous potential in science education as a fascinating model for learning about fundamental biological concepts, and in research for developing knowledge with medical applications for reproductive biology, embryonic development, and genetic interactions. These lizards maintain genome integrity in a hybrid state in which recombination is absent, but do not suffer from conditions or disorders such as Down's Syndrome or cancer that are caused by aneuploidy in humans. The multifarious impacts of hybridization on the diversity of species in this group present an exceptional opportunity to deepen understanding of the complicated process of evolutionary diversification.

*“We realize, however, that our colleagues may come, with Shakespeare, to feel that “ ’Tis the times” plague, when mad-men lead the blind’.”* –Duellman and Zweifel (1962) lamenting attempts to understand the evolutionary history of North American whiptail lizards

The science of biology is fascinating for its complexity and the understanding it provides about the diversity of living organisms that exist on earth. There are few taxonomic groups that exemplify this better than the North American whiptail lizards (*Aspidoscelis*; formerly included in *Cnemidophorus*), which are notable for being the group of vertebrates containing the largest diversity of unisexual species. Although previous authors have reviewed the potential for unisexual vertebrates to advance research in the life sciences (Neaves and Baumann 2010, Laskowski et al., 2019), less attention has been given to the opportunities these species provide to advance science education. The history of discovery in whiptail lizards represents a valuable case study on the progression of science, from the early studies on taxonomy and systematics, to the identification of unisexual species, and finally to the resolution of the mechanisms by which these species have formed and persisted. The whiptail lizard system also represents a fascinating framework in which to introduce a manifold of fundamental concepts in biology including meiosis, gene regulation, and development, due to the remarkable deviations that are found in these species. Whiptail lizards vividly illustrate many topics that are covered in introductory evolutionary biology courses, including the evolution of sex, where the presence of so many unisexual species represents an apparent paradox that simultaneously emphasizes the importance of genetic recombination provided by sex (i.e., the only extant unisexual species have formed in the very recent past). Mechanisms of microevolution and speciation in these lizards can also be used to develop active-learning modules in courses with real data to reinforce many of the most challenging concepts for undergraduate students to grasp, including species concepts, “tree-thinking” and phylogenetics, genome evolution (e.g., through polyploidization and mutation accumulation), and evolutionary ecology in the context of reproductive mode differences between species.

Despite these phenomena having inspired a large amount of research during the last 50 years, many non-specialists still struggle to understand the details of the system and the depth of insights that biologists have gained, limiting its value in education and research. This review seeks to summarize the history, breadth, and complexity of whiptail lizard research into a more understandable format to remedy these gaps and further promote the integration of these concepts into educational resources and research. Here, we focus on the ways in which the whiptail lizard system illustrates foundational concepts in biology that are of interest to a broad audience of scientists, educators, and citizens.

## History of research

**Figure 1.**
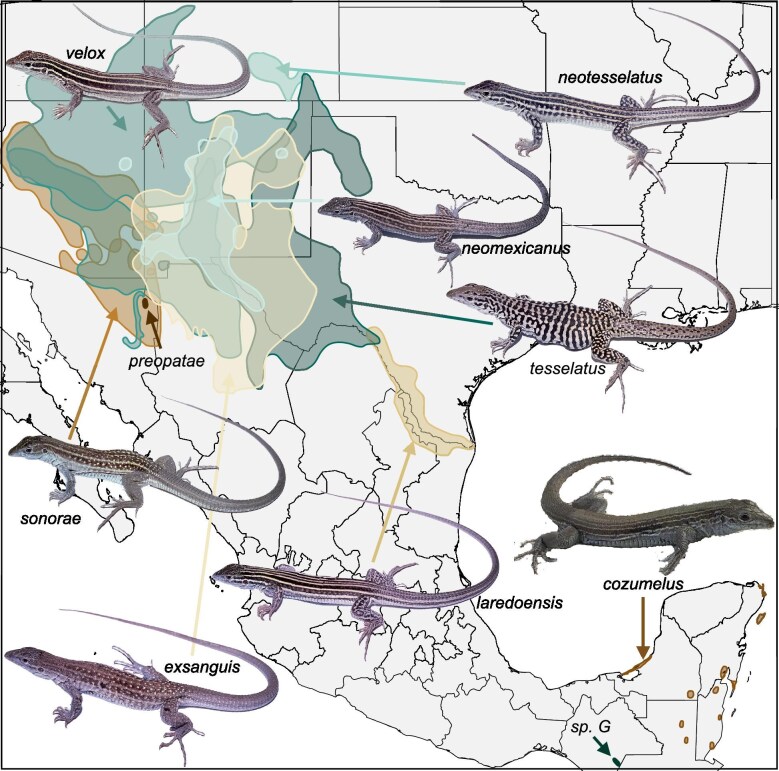
Map illustrating geographic distributions of unisexual species of *Aspidoscelis*. Photos of lizards by CJC and Fausto Mendez; distribution data from iucnredlist.org.

In the late 1950s, biologists presumed that all species of amniotic vertebrates had two sexes and reproduced through fertilization of egg cells by sperm (see Glossary text box for definitions of scientific terminology used in this manuscript). No alternative was considered given that all species that had been sufficiently studied were found to include both sexes, until a Russian herpetologist reported credibly on lacertid lizards in Armenia that had no males; unisexual females reproduced without them (reviewed by Darevsky 1966). Virtually all biologists were in disbelief, even after a few authors (Minton 1959, Duellman and Zweifel 1962, Maslin 1962) reported on the apparent lack of males also in several species of whiptail lizards (figure 1), a genus of about 50 species that occur in most of North America from Minnesota, United States, to Costa Rica (Wright 1993). These findings seemed incongruent with fundamental rules of biology across numerous subfields, ranging from developmental biology to population genetics. How would meiosis work? What would trigger embryonic development? Can unisexual species persist over time if they lack the benefits of genetic recombination provided by sex? How did they form in the first place given that this does not fit within foundational models for the process of species formation?

Biologists showed great resistance to the early reports of unisexuality and individuals who accepted them were thought to be misguided. As a graduate student of Bill Duellman's at the University of Kansas in 1963, CJC asked him what he thought about the all-female lizards discussed in the 1962 paper on which he was the lead author, and Duellman said “I don't believe that stuff, but I allowed it in the paper because Zweifel was so insistent on it!” Similarly, Tinkle (1959: 197) wrote “no explanation is advanced for the lack of males (in a series of 65 *A. tesselatus*) because it is felt that continued collecting of large series from all seasons will reveal the presence of males.” Despite this skepticism, numerous scientists were rewarded for their efforts to expand knowledge related to unisexuality in whiptails.

**Figure 2.**
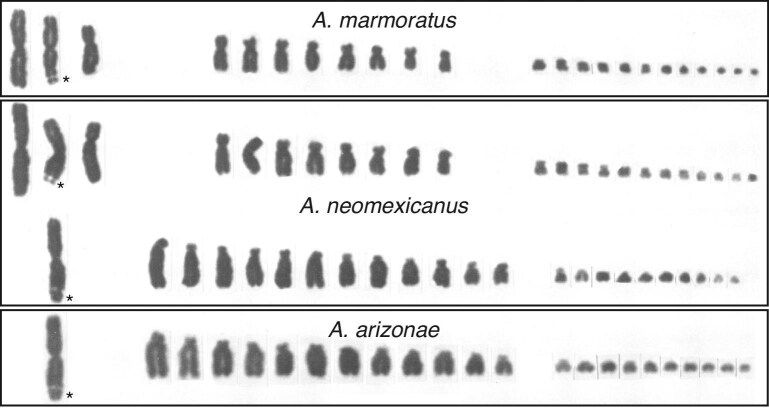
Diploid karyotype of a unisexual species of whiptail lizard (*A. neomexicanus*) and haploid karyotypes of its bisexual, parental progenitors *A. arizonae* and *A. marmoratus*. All these species possess the same number of chromosomes (2n = 46), but due to structural changes that evolved during the divergence between the bisexual species, chromosomes in the unisexual species can be readily assigned to each parent. For example, the two parental species differ in the number of macro vs. micro chromosomes, and one can identify that Chromosome 1 in *A. arizonae* has a longer terminal satellite than in the homologous Chromosome 2 in *A. marmoratus* (see asterisks at secondary constriction sites). Data from Cole et al. (1988).

Early studies on this topic produced chromosome spreads showing an abnormal number or extra set of homologous chromosomes in some unisexual species, which is largely unknown among bisexual vertebrates and can be indicative of the combining of genomes from divergent species in a single individual through hybridization (Pennock 1965, Lowe and Wright 1966, Lowe et al. 1970a,b). *These studies remain valuable today in education because they include karyotypes that allow students to “see” two or three genomes in a hybrid, unisexual species* (figure 2)*!* Around the same time, tissue histocompatibility studies found that unlike in sexual populations, different individuals in unisexual populations accepted reciprocal skin transplants (Maslin 1967, Cuellar 1976, Cordes and Walker 2006). This research demonstrated that individuals within these populations were genetically identical (or nearly so), confirming clonal reproduction and providing a pedagogical example of scientific hypothesis testing spanning multiple biological scales (i.e., illustrating the connection between genotype and phenotype). Parthenogenesis was later confirmed as the mode of reproduction in these lizards by histological examination of reproductive tissues across two generations, which found no evidence of sperm or testicular tissue, and ruled out hermaphroditism or sex reversal (Hardy and Cole 1981). Allozyme studies further confirmed the origins of these populations through hybridization by showing that individuals had very high levels of heterozygosity (figure 2; Neaves 1969, Parker and Selander 1975). In addition, the development of methods to maintain whiptail lizards in captivity was of basic importance for obtaining data from individuals of known genealogy through several generations (Townsend 1979, Townsend and Cole 1985).

In the 1970’s in Wesley M. Brown's laboratory at the University of Michigan, researchers first confirmed the maternal, sexual ancestor of several unisexual species by showing high similarity in (the maternally inherited) mitochondrial DNA between them (Brown and Wright 1979). Clonal reproduction in a laboratory colony of individuals later showed karyotypes and allozymes with preserved heterozygosity across many regions of the genome (Cole 1979, Dessauer and Cole 1986). Finally, the general mechanism by which unisexual whiptails produce egg cells was first identified in the 1970’s. After the offspring of parthenogenetic females were shown to be genetically identical to their mother, it was understood that there must be some modification to this process that allows them to produce egg cells containing the same ploidy and genome content, rather than only having half of her genome (as in sexual females). Cuellar (1971) demonstrated that this occurs because ovarian cells in these females enter meiosis with an extra set of chromosomes compared to what is normally observed, and still undergo the typical two rounds of cell division.

**Figure 3.**
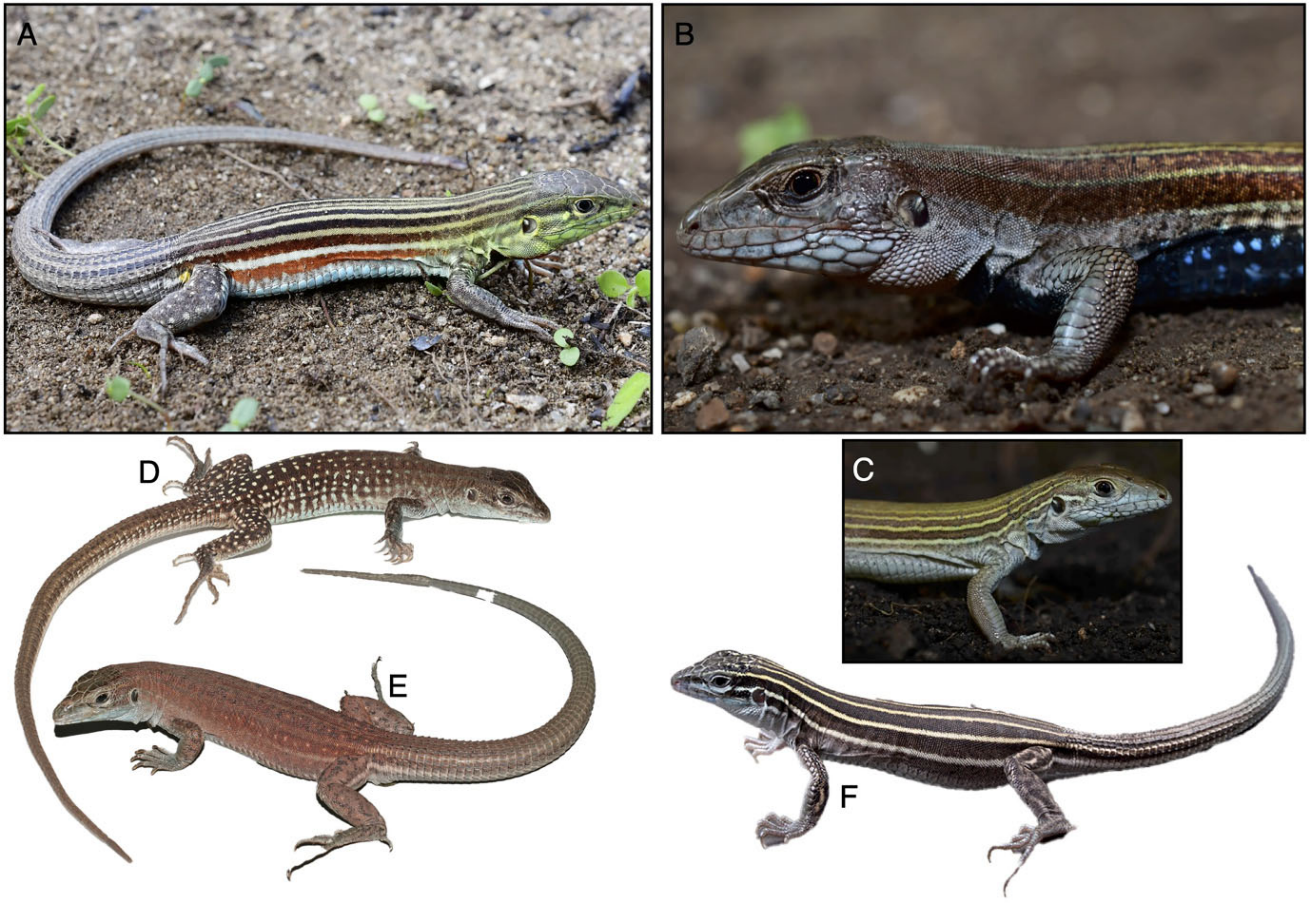
Photos of two bisexual species of *Aspidoscelis, A. deppii* (A) and *A. angusticeps* (B) that are the parental progenitors of the diploid, unisexual species *A. cozumelus* (C). The tetraploid unisexual species of *Aspidoscelis* are *A. townsendae* (D), *A. neavesi* (E), and *A. priscillae* (F). Photos by AJB, William Neaves, and Peter Baumann.

Among vertebrates, there are a few unisexual species of fishes, frogs, and salamanders with extraordinary, related modes of reproduction in which females require spermatozoa from males of a different, but related species to initiate development of their eggs (Neaves and Baumann 2010). These species vary in the extent to which the males make a genetic contribution to the resulting offspring (with their modes of reproduction being termed “gynogenesis” or “hybridogenesis”; see Box 1 in Neaves and Baumann 2010). However, individual females of parthenogenetic squamates (snakes and lizards) normally reproduce independently, cloning themselves in lineages with fixed heterozygosity generation after generation (although new mutations occur each generation as in sexual species). Whiptail lizards are generally abundant, conspicuous, wary, rapidly moving predators of arthropods (figure 3), called “Speedos” by some, “Racerunners” by others (the marbled whiptail lizards can sprint 26.0 km/hr, as fast as some Olympic runners, but with little endurance; Dohm et al. 1998). This makes many species exceptionally challenging to capture for study. Interestingly, the endurance capacity of some unisexual whiptails appears reduced in comparison to their bisexual ancestors (Cullum 1997), and these species are often more amendable to the lizard lassoing techniques frequently employed by herpetologists. The diversity of whiptail lizard species formed by different mechanisms makes them an exceptionally powerful model for testing theory related to biological processes that are widespread across the tree of life, but typically rare in any one taxonomic group (e.g., transitions in reproductive mode, hybridization, polyploidy).

Glossary Box

- **Allozyme:** different forms of proteins identified by gel electrophoresis.
- **Bisexual:** A species that includes individuals of two sexes. **Unisexual** species include only individuals of one sex (females).
- **Cloning:** reproduction that produces offspring that are genetically identical to the mother.
- **Karyotype:** the complete set of chromosomes of an individual.
- **Heterozygosity:** the presence of two different forms of a gene (i.e., allele) in a particular part of the genome of an individual.
- **Histocompatibility:** the degree of similarity between the major histocompatibility complex gene variants between cells, which often determine the success of organ and tissue transplants. These genes are typically highly variable among individuals in a population.
- **Hybridization:** the process of interbreeding between distinct species. This process can have a variety of evolutionary outcomes that depend on the reproductive capabilities of the hybrid offspring. Most commonly, repeated interbreeding between species and their fertile, hybrid offspring, over time can result in the exchange of alleles, a process termed **introgressive hybridization.**
- **Meiosis:** the cell division process that produces gametes, or sex cells (i.e., egg and sperm).
- **Parthenogenesis:** a form of asexual reproduction in which embryonic development proceeds from an unfertilized egg cell.
- **Ploidy:** the number of sets of homologous chromosomes within the cells of an organism. **Haploid** refers to the presence of a single set of chromosomes, **diploidy** to the presence of two sets (as in most vertebrates), and **polyploidy** to more than two sets, as in **triploidy** (three sets) or **tetraploidy** (four). **Aneuploidy** refers to an abnormal number of chromosomes.
- **Speciation:** the evolutionary process by which new species are formed. **Hybrid speciation** refers to a process in which hybridization is directly linked to the formation of a species.

## The unisexual species of whiptail lizards

**Figure 4.**
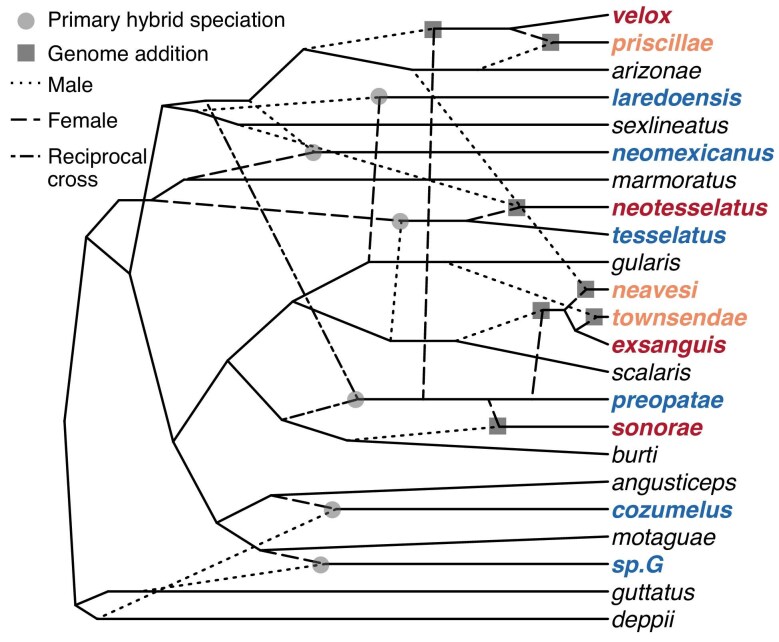
Evolutionary relationships among unisexual species of *Aspidoscelis* and the bisexual parental species from which they evolved through hybrid speciation. Diploid unisexual species (formed by primary hybrid speciation) are in blue, triploid unisexuals in red, tetraploid unisexuals in orange, and parental bisexual species in black (triploid and tetraploid unisexuals are formed by genome addition). Broken lines indicate hybridization edges with type indicating if the ancestral parent was male, female, or if the unisexual species is derived from reciprocal hybrid crosses.

Unisexual whiptail lizards vary in the number of sets of homologous chromosomes they possess, which reflects their history of hybridization and formation by two hybrid speciation mechanisms (figure 4; Barley et al. 2021b). Genome composition is of three basic kinds: diploid (as in sexual species, but including two divergent genome copies derived from different sexual species), triploid (sometimes including three distinct genome copies, or two of the three copies derived from the same sexual species), or tetraploid (often including aneuploidy, owing to new mutations; figure 3). Males do not exist and the females reproduce by parthenogenetic cloning. Rare instances of crossing between these females and males of other local, bisexual species sometimes produces triploid or tetraploid hybrid males or females (e.g., Cole et al. 2007), and this mechanism is how new species sometimes form through the addition of another set of chromosomes (when the hybrid females can reproduce parthenogenetically). Based on karyological study of the western whiptail lizard, sex determination in these lizards is thought to be XY (Cole et al. 1969, Bull 1978), and thus the hybrid males would be considered XXY or XXXY.

In “primary hybrid speciation,” diploid species are formed when the first-generation female hybrid offspring of a male and female lizard of distinct bisexual species begins reproducing by parthenogenetic cloning. *These hybridization events are remarkable because the bisexual species involved appear to have been separated by ∼15–25 million years of evolution (Barley et al.*  2022*)!* Polyploid species form through “genome addition,” when the cloned egg of a unisexual female is fertilized by a bisexual male lizard (of one of their parental or an entirely different species), and the resulting female offspring reproduces clonally, with an extra set of chromosomes (figure 4). Although triploid species are known from nature, tetraploid species are only known from hybridizing captive *Aspidoscelis*. Because some of the species involved in these laboratory crosses have overlapping distributions and are known to hybridize (i.e., some tetraploid hybrids of unknown reproductive status have been found in nature; Lowe et al. 1970b, Neaves 1971), it is plausible that they could have formed tetraploid species in nature at some point, but that the tetraploids have either not persisted or been overlooked by biologists due to their similar morphology to the triploid ancestor. In theory, diploid unisexual species could be reformed by “genome reduction” when a tetraploid individual produces diploid eggs (Lowe et al. 1970b). This meiotic reduction mechanism could be readily overlooked in nature if it does occur and has been documented in unisexual salamanders and fish (Bogart and Licht 1986, Alves et al. 2001). In all cases, the new unisexual species arise in a single generation through “hybrid speciation” in which mating between a male and female of two different bisexual species produce female offspring that clone themselves (but with any non-lethal mutations that may occur). Consequently, populations of unisexuals do not experience the rules of cohesion in population genetics that bisexual species do (Cole 1990, Frost and Hillis 1990).

This form of speciation is conceptually distinct from classical speciation in which new, bisexual species are formed by gradual divergence across many generations from a single common ancestor (de Queiroz 1998). This striking contrast is useful as an empirical example that can reinforce student understanding of mechanisms of microevolution and their importance to the process of speciation. It has also generated substantial philosophical disagreement among biologists on how to recognize species in unisexual vertebrates. Decades ago, CJC asked a prominent systematist about his opinion regarding the hybrid origins of unisexual whiptail lizards and he replied “this does not fit our ideas on speciation so we will ignore them!” Here, we propose that names for hybrid, unisexual species should be based on ancestry, with one specific name for clones of origin from each different combination of ancestral bisexual species. We do not apply a different name for clones that might have originated through more than one hybridization event of the same ancestors, nor for those that originated from reciprocal hybridization of the same parental species (although we summarize cross directionality for species known in nature below), without regard to how many F_1_ female hybrids might have produced populations of each unisexual species. Unstable taxonomies result when names are based on estimates of how many hybridization events might have been involved or postformational variant clones. Most of the unisexual species were first described based on morphology before their genetic ancestry was understood (and in many cases before they were known to be unisexual!), which also has contributed to significant historical taxonomic confusion. Nevertheless, detailed information on the evolutionary history and origins of these species, studies of compatibility of combinations of genomes, and studies of mechanisms of adaptation all contribute to fundamental scientific knowledge and the value of unisexual species in education.

The concept of “geographical parthenogenesis” was developed as a model to describe the phenomenon that unisexual species often appear to have ranges that are characteristically distinct from their sexual ancestors (Vandel 1928). These include encompassing larger areas, at higher elevations and latitudes, that have a history of disturbance (e.g., covered by glaciers during cooler periods). While some of these patterns do not clearly characterize whiptail lizards, hybridization appears to have often occurred in regions of transition between biological communities formed by shifting of habitats accompanying climate change (which may also shift species distributions). There are two geographic areas known to be inhabited by the unisexual species of whiptails: (a) the southwestern United States and northern Mexico; (b) southern Mexico and eastern Guatemala/Belize (figure 1). Many of the habitats in these regions in which the unisexual species predominate have been characterized as ecotonal, marginal, or perpetually disturbed, leading to the unisexual species being described as the equivalent of “ecological weeds” (though many areas of sympatry between sexual and unisexual species are known; Wright and Lowe 1968). The unisexual whiptails can be broadly characterized as morphologically intermediate between their sexual parental species, though exceptions to this pattern exist across traits and species (Cole et al. 1988, Barley et al. 2021b). Although the ecological niches of unisexual whiptails have been extensively studied and found to exhibit some differences from their parental ancestors (e.g., Case 1990), few general patterns have been identified that clearly match theoretical predictions about their breadth (e.g., being consistently narrower as a result of their limited genetic diversity, or broadly general as a result of their hybrid nature; Vrijenhoek and Parker 2009).

## The diploid unisexuals

*Aspidoscelis cozumelus*: the Cozumel racerunner occurs in the Yucatan Peninsula of Mexico, northern Guatemala, and Belize, and originated through hybridization between the Yucatan whiptail (*A. angusticeps*; ♀) and the blackbelly racerunner (*A. deppii*; ♂; figure 3). There are several variants based on morphology, karyotypes, histocompatibility, and mitochondrial DNA (including populations previously named as *A. maslini* and *A. rodecki*; Taylor et al. 2014).

*Aspidoscelis* “*sp. G*”: Wright (1993) discussed another related species, which he called “species G,” which will soon be described and named (Nieto-Montes de Oca et al., *in preparation*). The origin of this species, which occurs in southern Mexico and eastern Guatemala, was through hybridization between the Giant whiptail (*A. motaguae*; ♀) and the Mexican racerunner (*A. guttatus*; ♂; Moritz et al. 1992, Barley et al. 2022). To date, little is known about this lizard.

*Aspidoscelis laredoensis*: the Laredo striped whiptail occurs in southern Texas, the United States, and northern Mexico mostly in a narrow strip of land along the Rio Grande, and originated through hybridization between the eastern spotted whiptail (*A. gularis*; ♀) and the six-lined racerunner (*A. sexlineatus*; ♂). Data on morphology, karyotypes, allozymes and mitochondrial DNA (Wright et al. 1983) have revealed some variant clones thought to be derived from independent hybridization events (Barley et al. 2021a).

*Aspidoscelis neomexicanus*: the New Mexico whiptail occurs in New Mexico, western Texas, and northern Mexico mostly along the Rio Grande, with isolated populations in northern Arizona that may be introduced. Data on morphology, karyotypes, allozymes, and mitochondrial DNA show that this species originated through hybridization between the marbled whiptail (*A. marmoratus*; ♀) and the little striped whiptail (*A. arizonae*; ♂; Cole et al. 1988, Densmore et al. 1989a, Cole et al. 2010).

*Aspidoscelis preopatae*: the Rio Bavispe whiptail is a species whose existence was long speculated upon as being the hypothetical maternal ancestor of several triploid unisexual species that were known to occur in the southwestern United States and northern Mexico, but which was only recently demonstrated to occur in northern Mexico (Moritz et al. 1989, Densmore et al. 1989b, Barley et al. 2021b). Morphological, karyotypic, allozyme, and DNA sequence data show that this species was formed through reciprocal hybrid crosses between *A. arizonae* and the canyon spotted whiptail (*A. burti*).

*Aspidoscelis tesselatus*: the common checkered whiptail occurs in the southwestern United States and northern Mexico, and formed through hybridization between *A. marmoratus* (♀) and the rusty rumped whiptail (*A. scalaris*; ♂; Densmore et al. 1989a, Taylor et al., 2003, 2012, Barley et al. 2024). Some variants of this species were previously named as *A. dixoni* (Cole et al. 2007, de Quieroz et al. 2017). *Aspidoscelis tesselatus* is the maternal ancestor of *Aspidoscelis neotesselatus*, a triploid unisexual of hybrid origin. Frequent hybridization between female *A. tesselatus* and male *A. marmoratus* in southern New Mexico produces abundant triploid hybrids of both sexes, all or most of which appear to be sterile (Taylor et al. 2001).

## The triploid unisexuals

*Aspidoscelis velox*: the plateau striped whiptail occurs in northern Mexico and the southwestern U.S. and was formed through two steps of hybridization (Dessauer and Cole 1989, Moritz et al. 1989, Barley et al. 2021b). The first crosses were between *A. arizonae* and *A. burti*, which produced the diploid unisexual *A. preopatae* (♀), and triploids were formed through backcrosses between *A. preopatae* and a male *A. arizonae*. Populations described as *A. velox* (Springer 1928) are derived from an initial cross in which the female was *A. burti* and the male was *A. arizonae*, whereas populations later described as *A. uniparens* (Wright and Lowe 1965) and *A. opatae* (Wright 1967) were derived from reciprocal initial crosses between a male *A. burti* and a female *A. arizonae*. Because all lineages in this species are triploids that have two genomes from *A. arizonae* and one from *A. burti* we use the name *A. velox* for all of them.

*Aspidoscelis sonorae*: the Sonoran spotted whiptail occurs in southern Arizona and New Mexico in the United States and northern Mexico, and also originated through two steps of hybridization, with the first cross being between *A. arizonae* (♀) and *A. burti* (♂) producing the diploid unisexual *A. preopatae*. However, because in this case the second cross was between *A. preopatae* and a male *A. burti* (Dessauer and Cole 1989, Moritz et al. 1989, Barley et al. 2021b), individuals of this species have two genomes from *A. burti* and one from *A. arizonae*. This species has numerous diverse clones in morphology, karyotypes, and allozymes of unknown origins (Lowe et al. 1970b, Cole 1979, Dessauer and Cole 1989), one of which was previously recognized as *A. flagellicaudus* (Taylor et al. 2018).

*Aspidoscelis exsanguis*: another triploid species derived from *A. preopatae* is the Chihuahuan spotted whiptail, which occurs in southern Arizona, New Mexico, and western Texas in the United States and northern Mexico. In this case the first was the cross between a female *A. burti* and a male *A. arizonae* (Moritz et al. 1989), and the second cross was with a male *A. scalaris* (♂; Barley et al. 2021b, Cole et al. 2023, Barley et al. 2024). *As a consequence, individuals of this species have genomes that are derived from three different bisexual species!* Variant clones of karyotypes (Cole 1979), allozymes (Dessauer and Cole 1989), and morphology (Taylor et al. 2019) have been described.

*Aspidoscelis neotesselatus*: the Colorado checkered whiptail occurs in Colorado in the United States, and originated through two steps of hybridization. The first the cross between *A. marmoratus* (♀) and *A. scalaris* (♂) produced the diploid unisexual *A. tesselatus* (♀; Neaves 1969, Parker and Selander 1975, Barley et al. 2024). The second cross between *A. tesselatus* and *A. sexlineatus* (♂) resulted in *A. neotesselatus*. This species includes several clones of morphology and allozymes, but only one karyotype (Walker et al. 1997, Taylor et al. 2015, Taylor and Livo 2023).

## The tetraploid unisexuals

There are three described species of tetraploid parthenogenetic vertebrates (figure 3). All resulted from maintaining captive *Aspidoscelis* of two or more known species in enclosures at the Stowers Institute for Medical Research as part of a research initiative spearheaded by William B. Neaves. No experimental procedures were applied and the lizards underwent hybridization as whiptail lizards are known to do in nature, producing unisexual lineages of robust individuals that were maintained generation after generation through cloning of unfertilized eggs. Individuals of each of these species are extremely similar to the maternal ancestor of the cross.

*Aspidoscelis neavesi*: Neaves’ whiptail lizard resulted from the crossing of the triploid *A. exsanguis* (♀) x *A. arizonae* (♂). Consequently, this species has one haploid genome from *A. burti*, one from *A. scalaris*, and two from *A. arizonae*. Three hybrid females from two clutches of eggs founded new lineages from this cross (Cole et al. 2014). An adult female of this hybrid origin was found in nature (Neaves 1971) but it was not known whether it was an F_1_ hybrid or a clone.

*Aspidoscelis priscillae*: Priscilla's tetraploid whiptail lizards resulted from the crossing of *A. velox* (♀) x *A. arizonae* (♂). Consequently, this species has one haploid genome from *A. burti* and three from *A. arizonae*. At least 44 hybrid females created self-sustaining lineages from at least 15 clutches of eggs through this cross (Cole et al. 2017).

*Aspidoscelis townsendae*: Townsend's whiptail lizard resulted from the crossing of *A. exsanguis* (♀) x *A. gularis* (♂). Consequently, this species has a haploid genome from each of four species: *A. burti, A. arizonae, A. scalaris,* and *A. gularis*. At least two hybrid females from two clutches of eggs cloned lineages from this cross (Cole et al. 2023).

## Parthenogenetic reproduction in vertebrates

**Figure 5.**
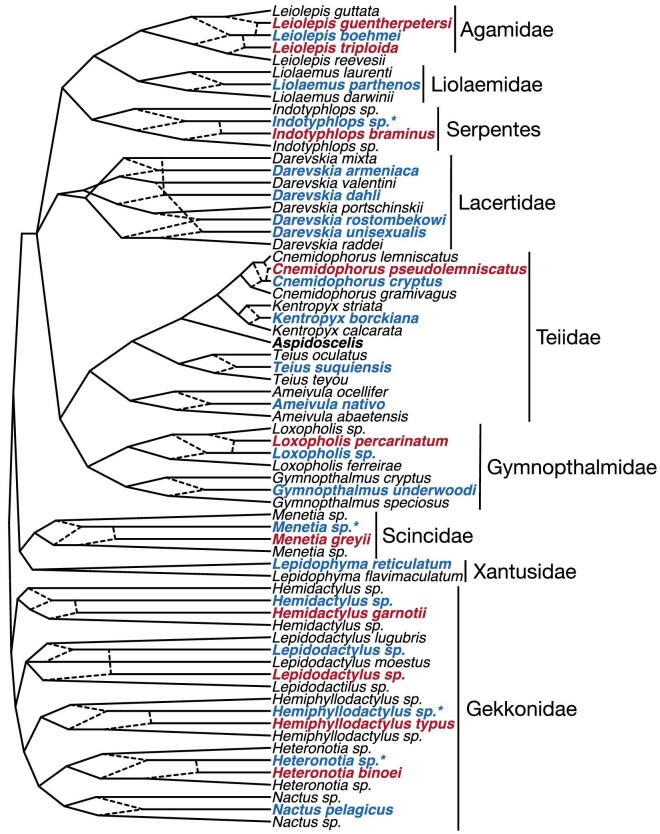
Hypothesized evolutionary relationships among the known obligate unisexual species of snakes and lizards (except *Aspidoscelis*, which are shown in figure 4) with Family names indicated on right. Diploid parthenogens are in blue, triploid parthenogens in red, and parental bisexual species in black (note that *L. flavimaculatum* includes both bisexual and unisexual populations). In some cases, taxonomy of parental lineages is poorly known, but all parthenogenetic species (except in *Lepidophyma*) are thought derived by hybridization between divergent bisexual species (indicated by dashed edges). Hypothesized, but currently unknown diploid ancestors are indicated by asterisks.

Following the discovery of parthenogenesis in *Aspidoscelis* and *Darevskia* (Lantz and Cyren 1936, Darevsky 1958), unisexual species were discovered in several additional groups of squamate reptiles (Cole 1975, Daresvsky et al. 1985). In total, these include at least 40 of the ∼12,000 species of reptiles, including representatives of nine different lizard families and one snake (figure 5; see Table S1 for references to the most recent publications and additional discussion). Where available, genetic evidence indicates that nearly all had a hybrid origin and that parthenogenesis sprang into being in one generation following hybridization between bisexual ancestral taxa. The one exception to this involves the night lizards (*Lepidophyma*), in which parthenogenetic populations are not thought to be of hybrid origin due to their very low heterozygosity (Sinclair et al. 2009).

Diploid parthenogenesis originated more than 20 times in reptiles (Table S1). Although we do not know what genetic factors lead to the capability of this phenomenon, recent evidence suggests that it almost exclusively evolves when hybridization occurs between highly divergent bisexual species (Barley et al. 2022). In contrast, repeated hybridization between more closely related bisexual species may commonly result in introgressive hybridization, and this appears to explain many of the historical difficulties in resolving the evolutionary history of whiptail lizards (including Duellman and Zweifel's invocation of King Lear that opens this manuscript). Rather than one or more new mutations having occurred numerous times during the evolutionary history of squamates to initiate parthenogenetic reproduction, it appears that the capability existed in genetic systems all the way back to the most recent common ancestor, nearly 200 million years ago (Reeder et al. 2002, Sites et al. 2011). One wonders how many of the taxa known only from fossils were parthenogenetic species!

Unlike in bisexual vertebrates which mostly have two sets of homologous chromosomes, polyploidy is common in unisexual species (approximately 40% of parthenogenetic reptiles have more than two sets of chromosomes). Because the formation of these species does not involve a transition in reproductive mode (these species reproduce as did their diploid maternal parthenogenetic ancestors), this form of speciation is probably more likely than primary hybrid speciation (and data from crossing experiments that resulted in tetraploid species support this). It has been proposed that polyploid species may benefit evolutionarily from the adaptive flexibility provided by additional genome copies (Dessauer and Cole 1984), however, their prevalence may also reflect a relaxation of constraints on polyploidy that is provided by the different mechanisms of meiosis in unisexual species compared to sexual species.

While primarily (a.k.a. obligate) unisexual vertebrates are rare, recent evidence suggests that an even broader range of primarily bisexual vertebrate species may rarely (facultatively) reproduce by parthenogenesis (including birds, sharks, and crocodilians; see supplemental material for a discussion of credible cases in reptiles). This phenomenon was initially noted in captive snakes after females that were caged in isolation for many years suddenly gave birth, but increasing evidence suggests that this mode of reproduction also occurs at a low level in many species in nature (Booth et al. 2012, Kratochvíl et al. 2020, Ho et al. 2024). This includes primarily bisexual species of whiptail lizards, however, the significance of this form of reproduction, if any, is unclear. This more labile view of vertebrate reproduction beckons additional investigation.

Study of the remarkable deviations that have evolved in unisexual vertebrates have enormous potential to advance scientific understanding of fundamental biological processes across a range of fields. This has become particularly apparent in recent years through studies of cell and genome biology in whiptail lizards and other unisexual vertebrates, which have generated insights into the mechanistic basis of reproductive mode variation. For example, fluorescent *in situ* hybridization techniques have shown that chromosome number and genetic diversity are maintained in unisexual whiptails over generations because identical, duplicated chromosomes (rather than homologous chromosomes) pair during meiosis (Lutes et al. 2010). Further, the vast majority of egg cells that enter meiosis in these females do not contain the necessary nuclear whole genome duplication, and as a consequence, the pairing of divergent homologous chromosomes fails (Newton et al. 2016). Surprisingly, even though only a relatively small proportion of cells with this duplication are able to progress through meiosis, this does not impair the fecundity or ability of these females to produce viable eggs.

More recently, genomic techniques were used to demonstrate that rare parthenogenetic reproduction in bisexual whiptail lizards proceeds quite differently: egg cells are haploid following meiosis as is typical, but diploidy is restored through failed cell division early in development (Ho et al. 2024). The deviations from canonical meiosis in whiptail lizards represent a unique evolutionary framework in which to introduce students to the different components of the cell division process and their biological importance (e.g., see figure 3 in Lutes et al. 2010 and figure 1 in Ho et al. 2024). Recent research has also identified the mechanism by which unisexual whiptail lizards maintain homogeneity of their ribosomes despite their genome being comprised of chromosomes from highly divergent species: selective inactivation and elimination of particular gene copies (Porter et al. 2019). This suggests that rather than being static as was historically assumed (Vrijenhoek 1989), the genomes of unisexual species may be quite dynamic, and capable of rapid evolution through gene conversion, chromosome structural changes, and epigenetic modifications that affect gene regulation (Majtánová et al. 2016, Warren et al. 2018, Jaron et al. 2020, Janko et al. 2021). The rapid advancement of genomic and biotechnology techniques will likely transform our understanding of the molecular basis of unisexual reproduction and its evolutionary outcomes in the near future.

## Supplementary Material

biaf010_Supplemental_File

## Author Biography

Anthony J. Barley (ajbarley@asu.edu) is an Assistant Professor of Biology in the School of Mathematical and Natural Sciences at Arizona State University–West Valley in Glendale, Arizona 85306 in the United States. Charles J. Cole (cole@amnh.org) is a Curator Emeritus with the Department of Herpetology at the American Museum of Natural History in New York, New York 10024, in the United States.
