# Supplementary material for: Speciation by hybridization: the mind-boggling nature, educational, and research value of the largest group of unisexual vertebrates
Source: Bioscience. 2025 Feb 14;75(4):331–41. doi: 10.1093/biosci/biaf010 (PMC12016801; doi:10.1093/biosci/biaf010)
Supplement: biaf010_Supplemental_File [file biaf010_supplemental_file.pdf]

Supplementary Material for: Speciation by hybridization: the mind-boggling nature, educational, and research value of the largest group of unisexual vertebrates.

Anthony J. Barley[a*] PhD, Charles J. Cole[b] PhD

a. School of Mathematical and Natural Sciences, Arizona State University–West Valley, Glendale, Arizona 85306, United States.

b. Department of Herpetology, American Museum of Natural History, Central Park West at 79th Street, New York, New York 10024, United States.

[*] **Corresponding author:** Anthony J. Barley @: ajbarley@asu.edu

Additional obligate parthenogenetic unisexual species of reptiles

Extensive evidence on obligate parthenogenesis in whiptail lizards followed initial suggestions of probable unisexual populations of *Darevskia* in Armenia (Lantz and Cyrén 1936, Darevsky 1958). Since then, reports of additional unisexual species of reptiles have appeared (Fig. 5; Supplemental Table 1). Evidence on the evolutionary origins and parthenogenesis in these species is extensive, but we cite only the most recent references with the most significant information from which the history of the science can be found. These publications provide the evidence of hybrid origins illustrated in Figure 5 and are summarized in Supplemental Table 1.

As with the unisexual *Aspidoscelis* and *Darevskia*, the recent research on the other unisexual lizards has revealed that nearly all of them had a hybrid origin and that triploid parthenogens resulted from hybridization between an ancestral diploid parthenogen and a bisexual species (the exception being in the genus *Lepidophyma* for which current evidence favors a non-hybrid origin of the parthenogenetic populations). For some species, such as

Heteronotia and *Gymnophthalmus*, the taxonomy of the cryptic species has not been resolved, and some of these species warrant additional comment.

Gymnophthalmus underwoodi: originally, the hybrid origin of this species was indicated by extremely high heterozygosity, but only one of the cryptic ancestors was identified genetically, while details of the second, cryptic, unnamed ancestor were hypothesized (Cole et al. 1990). Later, the second ancestor was found with the predicted karyotype and allozyme characters (Cole et al. 1993). Benozzati and Rodrigues (2003) confirmed evidence that the unisexual population on Ilha de Maraca, Brazil probably had an origin different from the populations in the Guianas (Cole et al. 1990). There probably are more cryptic species to be found in this complex.

Lepidophyma: Despite being similar in DNA sequences, enzymes, chromosomes, scalation, and color pattern, populations of *L. flavimaculatum* in Panama and most of Costa Rica lack males, in contrast to populations from further north in Central America and Mexico that contain them (Bezy and Camarillo 2002), suggesting that they likely represent two species. The related species *L. reticulatum* from Costa Rica also appears to consist of only female individuals. These species are unique among the obligate parthenogenetic species in that there is no evidence indicating that they had a hybrid origin, as these species do not show elevated heterozygosity (and unisexuality may have evolved independently in each lineage; Sinclair et al. 2009). However, mixoploidy was also found in unisexual *L. flavimaculatum* from Panama (Bezy 1972). It is conceivable that in the parthenogenetic *L. flavimaculatum* and *L. reticulatum* there was an original hybridization event, but the hybrid females produced eggs in a way that essentially eliminates heterozygosity and introduces mixoploidy, and thus we suggest that this hypothesis should be further examined.

Hemidactylus garnotii: this is a complex of diploid and triploid cryptic unisexual forms in Thailand and the Indo-Pacific that might have had origins from hybridization among cryptic species (Ota et al. 1996).

Lepidodactylus lugubris: this species of uncertain taxonomic status is widespread in the tropics, consists of 2n and 3n populations, and there are several cryptic species among the bisexual ancestors of the unisexuals. The type specimen is thought to refer to a diploid, bisexual population (Robert Fisher, *personal communication*), so we refer to the unisexuals as "*sp.*".

Heteronotia binoei: this is a complex of morphologically similar species of geckoes that are widespread in Australia and include extensive genetic variation. Two undescribed bisexual forms have been identified as the ancestors of the triploid unisexual forms. Data support the conclusion that diploid, parthenogenetic hybrids backcrossed with both parentals to form a triploid parthenogenetic species, but the diploid parthenogenetic forms have not been found (Moritz 1993; Moritz and Heideman 1993).

Nactus pelagicus: this complex of geckoes, including bisexual and unisexual species, is widely distributed in Australia and on islands of the South Pacific. Extensive genetic data suggest that the parthenogens are diploid and had at least one hybrid origin. Populations representing a good candidate for the paternal ancestor have been identified, but the maternal ancestor remains unknown (Donnellan and Moritz 1995, Zug and Moon 1995).

Hemiphyllodactylus typus: this represents one of the most poorly known complexes of bisexual and unisexual cryptic species of geckoes, which occur in the Indo-Pacific. Nevertheless, Dedukh et al. (2022) demonstrated that the unisexual species is triploid and has chromosomes representing two or more ancestral species.

Facultative parthenogenesis in other reptiles

Among reptiles, on rare occasions a female of a normally bisexual species will produce an embryo or living offspring from an apparently unfertilized egg (called facultative parthenogenesis or FP). Individuals developed through FP often are abnormal or inviable, but at times apparently normal offspring are produced. This was initially noted more than six decades ago in captive snakes that had been kept in isolation for years. It was then thought that fertilization of eggs occurred by spermatozoa that had been stored for years in the female's spermathecae, as development without fertilization was not believed to be possible. However, increasing evidence indicates that FP occurs at a low frequency in many species of snakes, lizards, and a crocodile, not only in captivity, but also sometimes in nature (Booth et al. 2012, Kratochvil et al. 2020, Ho et al. 2024). The evolutionary significance of this, if any, is unknown and warrants additional investigation. We say "if any" because the production of a vertebrate ovum involves a very complicated procedure, and it should not be surprising if in some cases mistakes occur. Facultative parthenogenesis has not been documented in mammals, presumably due to the barrier of genomic imprinting. Parthenogenetic events in humans have been documented in producing ovarian tumors, although no viable offspring have been reported with support of credible genetic evidence (Carli and Pereira 2017).

In many instances of unexpected reproduction in captive reptiles evidence for FP or delayed fertilization is equivocal. Here we review only those reports in which significant genetic evidence for FP has been documented. Genetic evidence includes analyses of microsatellite DNA (msDNA) and nuclear DNA sequences, in which the mother is compared with her offspring and other individuals. In most cases, the method of formation of the egg by the female remains

uncertain, but in general, FP-produced embryos appear to be formed by a method different from those of obligatory parthenogenetic species (Ho et al. 2024).

Booth et al. (2023) reported FP in *Crocodylus acutus* after a captive female produced a clutch of 14 eggs. The female had been obtained at two years of age and housed without contact with other crocodilians for 16 years before oviposition. One egg developed into a fully formed fetus that failed to hatch. If the genetic basis for FP among the modern reptiles extends back through the reptilian phylogeny, this suggests it existed on the order of 280 million years ago. FP was reported in *Varanus panoptes* (family Varanidae) by Lenk et al. (2005); in *Varanus komodoensis* by Watts et al. (2006); in *Physignathus cocincinus* (family Agamidae) by Miller et al. (2019); in *Lepidophyma smithi* (family Xantusiidae) by Kratochvil et al. (2020); and in *Aspidoscelis arizonae* and *Aspidoscelis marmoratus* (family Teiidae) by Ho et al. (2024).

In *Aspidoscelis*, FP-developed embryos or offspring showed homozygosity, including at loci where the mother was heterozygous. Mixoploidy (presence of haploid cells and diploid cells in an individual) suggested that development began in a haploid unfertilized post-meiotic oocyte (not resulting from cell fusion) and later diploidy resulted from failed cytokinesis in some cell lines. Of at least 23 incidents of FP, only 14 eggs hatched and most individuals had developmental defects. One female of *A. arizonae* produced a clutch of eggs with some offspring resulting from FP, others from fertilized eggs. In addition, genome sequencing of many individuals of 15 species of *Aspidoscelis* sampled in natural populations revealed five with essentially total homozygosity that may have resulted from FP (5 of 231 lizards, or 1.6%). Frequency of FP in captive *A. arizonae* was estimated to be about 1%, but 5% in *A. marmoratus* (Ho et al. 2024).

In *Lepidophyma smithi* six females aged one month were raised and after three years all produced offspring having had no contact with males. Male offspring and female offspring were produced by FP; sexually-produced females can reproduce by FP; FP-produced females reproduced either sexually or by FP; and FP-produced males were fertile. The authors suggested that cryptic FP may be occurring in nature in species of *Lepidophyma* in which males occur (Kratochvil et al. 2020).

Booth and Schuett (2016) reviewed the literature of FP in snakes for examples that had been supported by significant genetic analyses. In addition to *Indotyphlops braminus* (the only obligatory parthenogenetic snake), they cited FP that occurred in 20 species representing 6 families throughout the phylogenetic diversity of snakes. In most cases near term embryos or recent hatchlings were studied. In boas and pythons, FP resulted only in female offspring many of which were viable and apparently normal, but in other snakes FP resulted only in males in clutches with very low viability and severe deformities in many individuals.

Booth et al. (2012) reported FP in nature in two species of snakes. For *Agkistrodon contortrix*, litters of 22 wild-caught pregnant females were produced in captivity, and for A*gkistrodon piscivorus*, litters of 37 wild-caught pregnant females. For both species, only one litter, which was small, had nearly all eggs fail, but one male appeared normal in each, and they were still growing and appeared normal at publication. These observations inspired significant DNA analyses of the litters, which indicated FP reproduction, including of the two survivors.

Shibata et al. (2017) reported FP in a captive green anaconda (*Eunectes murinus*), which produced offspring by sexual reproduction shortly after capture. After seven years in isolation from males, the female died of pneumonia at which time her oviducts contained 17 undeveloped eggs plus two essentially fully developed but dead female fetuses. Analyses of msDNA showed

that the fetuses inherited alleles present only in the mother, and assuming that they were diploid, all loci in the homozygous state.

Cubides-Cubillos et al. (2020) reported FP in three species of pit viper: *Bothrops atrox, Boothrops moojeni,* and *Bothrops leacurus*. A total of four litters were reported, in all of which many eggs showed no development and some embryos were abnormal or died just prior to birth, except for one living and apparently normal male offspring. Four msDNA loci showed that offspring were homozygous at all loci, including those at which the mother was heterozygous, and all alleles of the offspring were alleles found in the mother.

Allen et al. (2018) reported FP in two species of snakes in the family Elapidae (*Oxyuranus scutellatus* and *Acanthophis antarcticus*). For both species few eggs developed, some produced still born and abnormal offspring, but a total of three apparently normal male offspring were produced, with at least one living to an age of 3.5 years. The authors used ddRAD-seq nuclear SNP markers of the mothers and available offspring. Homozygosity at loci was very high in the offspring, but whether they had only alleles that occurred in their mothers was not stated.

Card et al. (2021) reported FP in the king cobra (Elapidae). In a clutch of 24 eggs only 2 were judged as viable and therefore incubated. Later, both were opened prior to hatching. One contained a dead essentially term fetus, the other contained a living fetus that died shortly thereafter. Both were males with craniofacial abnormalities, as has been seen previously in FP offspring of modern species of snakes. Genome-wide analyses of SNPs (thousands of loci) showed that the offspring had significantly reduced heterozygosity compared to the mother, also similar to FP in other modern snakes.

Supplementary References not included in main manuscript

Abdala CS, Baldo D, Juárez RA, Espinoza RE. 2016. The first parthenogenetic pleurodont Iguanian: a new all-female *Liolaemus* (Squamata: Liolaemidae) from western Argentina. *Copeia* 2016: 487–497.

Allen L, Sanders KL, Thomson VA. 2018. Molecular evidence for the first records of facultative parthenogenesis in elapid anakes. *Royal Society Open Science* 5: 171901. doi.org/10.1098/rsos.171901.

Adams M, Foster R, Hutchinson MN, Hutchinson RG, Donnellan SC. 2003. The Australian scincid lizard *Menetia greyii*: a new instance of widespread vertebrate parthenogenesis. *Evolution* 57: 2619–2627.

Arakelyan M, Spangenberg V, Petrosyan V, Ryskov A, Kolomiets O, Galoyan E. 2023. Evolution of parthenogenetic reproduction in Caucasian rock lizards: a review. *Current Zoology* 69: 128–135.

Benozzati ML, Rodrigues MT. 2003. Mitochondrial restriction-site characterization of a Brazilian group of eyelid-less gymnophthalmid lizards. *Journal of Herpetology* 37: 161–168.

Bezy RL. 1972. Karyotypic variation and evolution of the lizards in the family Xantusiidae. *Contributions in Science, Natural History Museum, Los Angeles County, CA* 227: 1–29.

Bezy, R. L., and J. L. Camarillo. 2002. Systematics of xantusiid lizards of the genus *Lepidophyma*. Contrib. Sci. LACM 493:1–41.

Booth W, Schuett GW. 2016. The emerging phylogenetic pattern of parthenogenesis in snakes. *Biological Journal of the Linnean Society* 118: 172–186.

Booth W, Levine BA, Corush JB, Davis MA, Dwyer Q, Plecker RD, Schuett GW. 2023. Discovery of facultative parthenogenesis in a new world crocodile. *Biology Letters* (2023)19. doi.org/10.1098/rsbl.2023.0129

Brunes TO, da Silva AJ, Marques-Souza S, Rodrigues MT, Pellegrino KCM. 2019. Not always Young: the first vertebrate ancient origin of true parthenogenesis found in an Amazon leaf litter lizard with evidence of mitochondrial haplotypes surfing on the wave of a range expansion. *Molecular Phylogenetics and Evolution* 135: 105–122.

Card DC, Vonk FJ, Smalbrugge S, Casewell NR, Wüster W, Castoe TA, Schuett GW, Booth W. 2021. Genome-wide data implicate terminal fusion automixis in king cobra facultative parthenogenesis. *Scientific Reports*, 7271. doi.org/10.1038/s41598-021-86373-1.

Carli GJd, Pereira TC. 2017. On human parthenogenesis. *Medical Hypotheses*106 (2017): 57–60. doi.org/10.1016/j.mehy.2017.07.008.

Cole CJ, Dessauer HC, Townsend CR, Arnold, MG. 1990. Unisexual lizards of the genus *Gymnophthalmus* (Reptilia: Teiidae) in the Neotropics: genetics, origin, and systematics. *American Museum Novitates* 2994: 1–29.

Cole CJ, Dessauer HC. 1993. Unisexual and bisexual whiptail lizards of the *Cnemidophorus lemniscatus* complex (Squamata: Teiidae) of the Guiana Region, South America, with descriptions of new species. *American Museum Novitates* 3081: 1–30.

Cole CJ, Dessauer HC, Markezich AL. 1993. Missing link found: the second ancestor of *Gymnophthalmus underwoodi* (Squamata: Teiidae), a South American unisexual lizard of hybrid origin. *American Museum Novitates* 3055: 1–13.

Cole CJ, Dessauer HC, Townsend CR, Arnold MG. 1995. *Kentropyx borckiana* (Squamata: Teiidae): a unisexual lizard of hybrid origin in the Guiana Region, South America. *American Museum Novitates* 3145: 1–23.

Cubides-Cubillos SD, Patané JSL, da Silva KMP, Almeida-Santos SM, Polydoro DS, Galassi GG, Cardoso SRT, Silva MJdeJ. 2020. Evidence of facultative parthenogenesis in three Neotropical pitviper species of the *Bothrops atrox* group. *PeerJ* 8:e10097. doi.org/10.7717/peerj.10097.

Dedukh D, Altmanova M, Klíma J, Kratochvíl L. 2022. Premeiotic endoreplication essential for obligate parthenogenesis in geckos. *Development (2022)* 149: 1–13 + supplementary information. Doi: 10.1242/dev.200345

Donnellan SC, Moritz C. 1995. Genetic diversity of bisexual and parthenogenetic populations of the tropical gecko *Nactus pelagicus* (Lacertilia: Gekkonidae). *Herpetologica* 51: 140–154.

Espeche BA, Brigada AM, Rivera PC. 2023. Morphometric variability in lizards of the genus *Teius*: a comparative study of species with different reproductive modes. *Journal of Herpetology* 57: 238–245.

Grismer JL, Bauer AM, Grismer LL, Thirakhupt K, Aowphol A, Oaks JR, Wood PL Jr., Onn CK, Thy N, Cota M, Jackman T. 2014. Multiple origins of parthenogenesis, and a revised species phylogeny for the Southeast Asian butterfly lizards, *Leiolepis*. *Biological Journal of the Linnean Society* 113: 1080–1093.

Ho DV, Tormey D, Odell A, Newton A A, Schnittker RR, Baumann DP, Neaves WB, Schroeder MR, Sigauke RF, Barley AJ, Baumann P. 2024. Post-meiotic mechanism of facultative parthenogenesis in gonochoristic whiptail lizard species. *eLife* 2024;0:e97035.

doi.org/10.7554/eLife.97035.

Karin BR, Oliver PM, Stubbs AL, Arifin U, Iskandar DT, Arida E, Oong Z,McGuire JA, Kraus F, Fujita MK, Ineich I, Ota H, Hathaway SA, Fisher RN. 2021. Who's your daddy? On the identity and distribution of the paternal hybrid ancestor of the parthenogenetic gecko *Lepidodactylus lugubris* (Reptilia: Squamata: Gekkonidae). *Zootaxa* 4999: 87–100.

Lantz, Louis Amédée, and O. Cyrén. "Contribution à la connaissance de Lacerta saxicola Eversmann." *Bulletin de la Société zoologique de France* 61 (1936): 159-181.

Lenk P, Eidenmueller B, Staudter H, Wicker R, Wink M. 2005. A parthenogenetic *Varanus*. *Amphibia-Reptilia* 26: 507–514.

Miller KL, Rico SC, Muletz-Wolz CR, Campana MG, McInerney N, Augustine L, Frere C, Peters AM, Fleischer RC. 2019. Parthenogenesis in a captive Asian water dragon (*Physignathus cocininus*) identified with novel microsatellites. *PLoS ONE* 14(6): e0217489. doi.org/10.1371/journal.pone.0217489.

Moritz C. 1993. The origin and evolution of parthenogenesis in the *Heteronotia binoei* complex; synthesis. *Genetica* 90: 269–280.

Moritz C, Heideman A. 1993. The origin and evolution of parthenogenesis in *Heteronotia binoei* (Gekkonidae): reciprocal origins and diverse mitochondrial DNA in western populations. *Systematic Biology* 42: 293–306.

Murphy RW, Fu J, MacCulloch RD, Darevsky IS, Kupriyanova LA. 2000. A fine line between sex and unisexuality: the phylogenetic constraints on parthenogenesis in lacertid lizards. *Zoological Journal of the Linnean Society* 130: 527–549.

Ota H, Hikida T, Matsui M, Mori A, Wynn AH. 1991. Morphological variation, karyotype and reproduction of the parthenogenetic blind snake, *Ramphotyphlops braminus*, from the insular region of East Asia and Saipan. *Amphibia-Reptilia* 12: 181–193.

Ota H, Hikida T, Matsui M, Chan-Ard T, Nabhitabhata, J. 1996. Discovery of a diploid population of the *Hemidactylus garnotii-vietnamensis* complex (Reptilia: Gekkonidae). *Genetica* 97: 81–85.

Radtkey RR, Donnellan SC, Fisher RN, Moritz C, Hanley KA, Case TJ. 1995. When species collide: the origin and spread of an asexual species of gecko. *Proceedings of the Royal Society of London B* 259: 145–152.

Rocha CFD, Bergallo HG, Peccinini-Seale D. 1997. Evidence of an unisexual population of the Brazilian whiptail lizard genus *Cnemidophorus* (Teiidae), with description of a new species. *Herpetologica* 53: 374–382.

Shibata H, Sakata S, Hirano Y, Nitasaka E, Sakabe A. 2017. Facultative parthenogenesis validated by DNA analyses in the green anaconda (*Eunectes murinus*). *PLoS One* 12: e0189654. doi.org/10.1371/journal.pone.0189654.

Watts PC, Buley KR, Sanderson S, Boardman W, Ciofi C, Gibson R. 2006. Parthenogenesis in Komodo dragons. *Nature* 444: 1021–1022. doi:10.1038/nature4441021a.

Wickramasinghe N, Wickramasinghe LJM, Vidanapathirana DR, Tennakoon KH, Samarakoon SR, Gower DJ. 2022. A molecular-genetics perspective on the systematics of the flowerpot blindsnake *Indotyphlops braminus* (Daudin, 1803) (Squamata: Serpentes: Typhlopidae). *Systematics and Biodiversity* 20: 1–16.

Zug GR, Moon BR. 1995. Systematics of the Pacific slender-toed geckos, *Nactus pelagicus* complex: Oceania, Vanuatu, and Solomon Islands populations. *Herpetologica* 51: 77–90.

Table S1. Obligate parthenogenetic species of Squamata. N indicates ploidy, H indicates hybrid origin (yes (Y) or no (N)), question marks indicate substantial uncertainty about these parameters.

Species	N; H	Family	Distribution	Ref.
Leiolepis boehmei	2n; Y	Agamidae	SE Asia	Grismer et al. 2014
Leiolepis triploida	3n; Y	Agamidae	SE Asia	Grismer et al. 2014
Liolaemus parthenos	3n; Y?	Liolaemidae	Argentina	Abdala et al. 2016
Indotyphlops braminus	3n; ?	Typhlopidae	Pantropical	Wickramasinghe et al. 2022
Darevskia armeniaca	2n; Y	Lacertidae	Caucasus	Murphy et al. 2000, Arakelyan et al. 2023
D. dahli	2n; Y	Lacertidae	Caucasus	Murphy et al. 2000, Arakelyan et al. 2023
D. rostombekowi	2n; Y	Lacertidae	Caucasus	Murphy et al. 2000, Arakelyan et al. 2023
D. unisexualis	2n; Y	Lacertidae	Caucasus	Murphy et al. 2000, Arakelyan et al. 2023

Aspidoscelis exsanguis	3n; Y	Teiidae	N. America	Barley et al. 2021b
A. sonorae	3n; Y	Teiidae	N. America	Barley et al. 2021b
A. preopatae	2n; Y	Teiidae	Mexico	Barley et al. 2021b
A. velox	3n; Y	Teiidae	N. America	Barley et al. 2021b
A. laredoensis	2n; Y	Teiidae	N. America	Barley et al. 2021a
A. neomexicanus	2n; Y	Teiidae	N. America	Cole et al. 2010
A. neotesselatus	3n; Y	Teiidae	Colorado	Taylor et al. 2015
A. tesselatus	2n; Y	Teiidae	N. America	Taylor et al. 2003
A. cozumelus	2n; Y	Teiidae	Mexico	Taylor et al. 2014
A. sp. G	2n; Y	Teiidae	N. America	Wright 1993
A. neavesi	4n; Y	Teiidae	Captivity	Cole et al. 2014
A. priscillae	4n; Y	Teiidae	Captivity	Cole et al. 2017
A. townsendae	4n; Y	Teiidae	Captivity	Cole et al. 2023
Cnemidophorus cryptus	2n; Y	Teiidae	S. America	Cole and Dessauer 1993
C. pseudolemniscatus	3n; Y	Teiidae	S. America	Cole and Dessauer 1993
Ameivula nativo	?; ?	Teiidae	Brazil	Rocha et al. 1997
Kentropyx borckiana	2n; Y	Teiidae	S. America	Cole et al. 1995
Teius suquiensis	2n?; Y?	Teiidae	Argentina	Espeche et al. 2023
Loxopholis sp.	2n;Y?	Gymnophthalmidae	S. America	Brunes et al. 2019

Loxopholis percarinatum	3n; Y?	Gymnophthalmidae	S. America	Brunes et al. 2019
Gymnophthalmus underwoodi	2n; Y	Gymnophthalmidae	S. America	Cole et al. 1993
Menetia greyii	3n; Y	Scincidae	Australia	Adams et al. 2003
Lepidophyma reticulatum	2n; N	Xantusiidae	Costa Rica	Sinclair et al. 2009
L. flavimaculatum	2n; N	Xantusiidae	Costa Rica and Panama	Sinclair et al. 2009
Hemidactylus sp.	2n; Y?	Gekkonidae	Thailand	Ota et al. 1996
Hemidactylus garnotii	3n; Y?	Gekkonidae	Indo-Pacific	Ota et al. 1996
Lepidodactylus sp.	2n; Y	Gekkonidae	Tropics	Radtkey et al. 1995
Lepidodactylus sp.	3n; Y	Gekkonidae	Tropics	Karin et al. 2021
Heteronotia binoei	3n;Y	Gekkonidae	Australia	Moritz 1993
*Heteronotia s*p.	2n?; Y	Gekkonidae	Australia	Moritz 1993; theoretical
Nactus pelagicus	2n; Y	Gekkonidae	Australia and S. Pacific	Donnellan and Moritz 1995
Hemiphyllodactylus typus	3n?; Y	Gekkonidae	Indo-Pacific	Dedukh et al. 2022
